# Posterior dislocation of the elbow as an unusual presentation after a total hip replacement: a case report

**DOI:** 10.1186/1752-1947-2-38

**Published:** 2008-02-06

**Authors:** Kumar Periasamy, Dominic Meek, Paul Crossman

**Affiliations:** 1 Department of Orthopaedics and Trauma surgery, Southern General Hospital, Glasgow, UK

## Abstract

**Introduction:**

Posterior dislocation of the elbow is usually associated with trauma to the joint with a reported incidence of 3%to 6%. Chronic instability is usually symptomatic following the initial injury.

**Case presentation:**

We report a case of posterior dislocation of the elbow occurring in a patient while using her arm to lift herself using a monkey pole on the second day following a total hip replacement. The dislocation was reduced under sedation in the ward. There were no signs or symptoms suggesting any joint hypermobility syndrome in the patient. Follow up 4 months following the injury revealed a complete recovery in the range of motion and a pain free elbow. There were no signs and symptoms of any instability.

**Conclusion:**

This is the first time such a case is reported in the literature. It certainly demonstrates that even in the absence of instability a patient can be predisposed to low energy dislocation of the elbow.

## Introduction

Posterior dislocation of elbow is infrequent but not uncommon [1]. Elbow dislocation is the second most common type of dislocation encountered in the adult population. The majority of these can be treated by closed manipulation and relocation [2]. It is well recognized that simple dislocations are commonly associated with good outcomes after a closed reduction and institution of early motion [3].

We report an unusual case of posterior dislocation of an elbow in an adult while trying to get out of bed using a monkey pole on the second post operative day after a hip replacement.

This is the first case to be reported in the literature with such an unusual mechanism

## Case presentation

**Figure 1:**
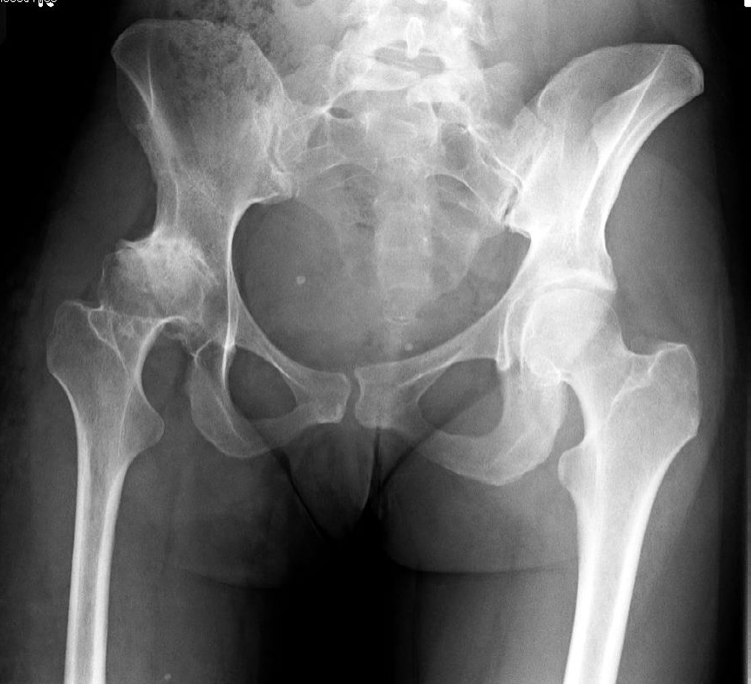
Pre op Pelvis.

**Figure 2:**
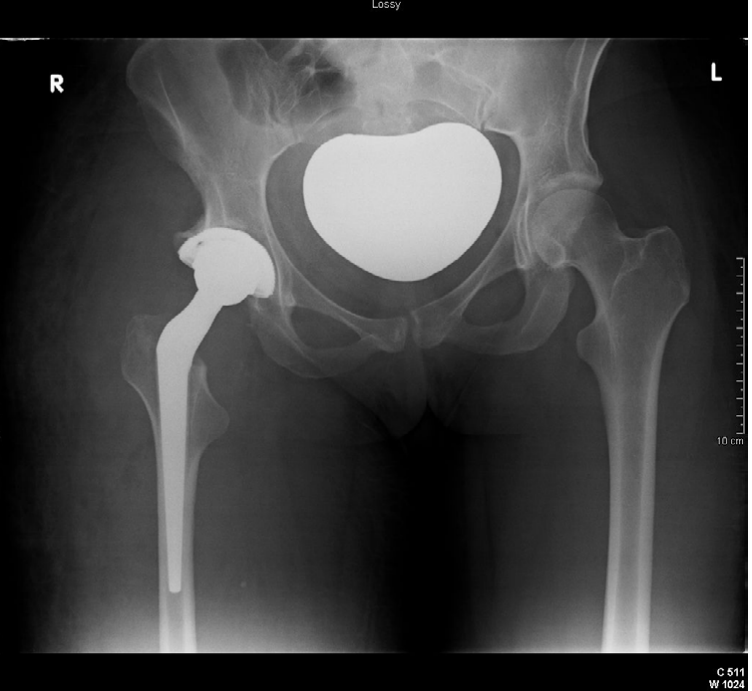
Post op Pelvis.

A 27 year woman, previously fit and healthy, who has congenital arteriovenous malformations localised to the right lower limb was seen in the orthopaedic clinic with a painful right hip. Assement of her hip confirmed a severely arthritic hip with radiological features of secondary arthritis associated with developmental dysplasia of the hip joint. She underwent an elective total hip replacement (Figure 1 and 2).

Surgery was performed routinely. While undertaking standard mobilisation, the patient was trying to get out of her bed on the second day post operation, heared a pop and presented with a painful deformed elbow on the left side

Patient was reviewed by the upper limb consultant who was on call. A posterior elbow dislocation was diagnosed and reduced on the ward under intravenous sedation. X ray of the dislocated elbow was not performed in view of the pain and the obvious clinical appearance confirming the diagnosis.

**Figure 3:**
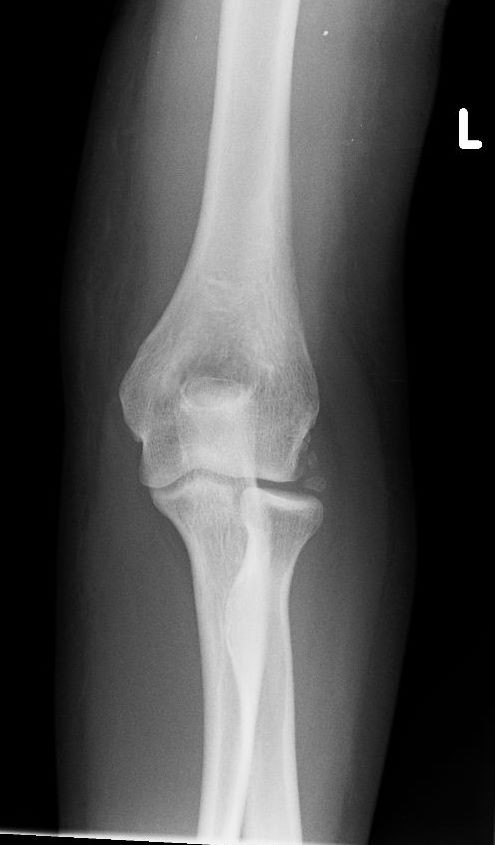
AP xray Elbow.

**Figure 4:**
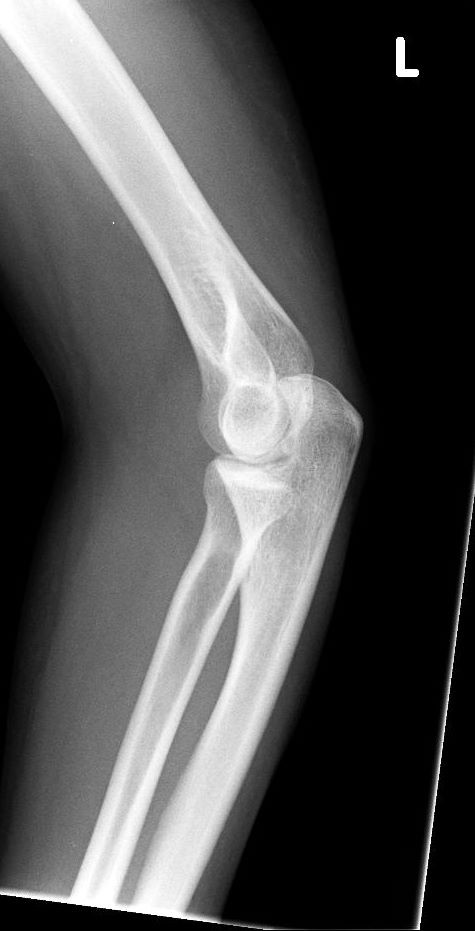
Lateral xray Elbow.

A check x-ray following the reduction confirmed relocation of the elbow joint and minor ectopic calcification in the lateral joint region of the elbow (Figure 3 and 4)

On further questioning the patient and her parents, they recollected that the patient had dislocated the same elbow at an age of 9 years and was reduced in the Accident and Emergency.

She did not have any residual effect of elbow instability prior to the present episode of dislocation and any evidence of generalised ligamentous laxity.

## Discussion

Elbow dislocation is not a frequently seen condition and is usually associated with significant trauma as it is an inherently stable joint. When an isolated dislocation of the elbow occurs without fracture, closed reduction of the elbow with early mobilization (within 2 weeks) results in a stable joint with good functional outcome [4].

With regard to the anatomy of the elbow, stability is afforded by both static and dynamic structures. Static structures include the complex bony architecture and soft-tissue stabilizers. The static soft-tissue stabilizers consist of the anterior and posterior joint capsule and both medial and lateral collateral ligament complexes. Of the ligaments around the elbow the lateral collateral ligament complex (LCLC) is defined as the Lateral collateral ligament (LCL) with its ulnar and radial components and the annular ligament. The lateral ulnar collateral ligament (LUCL) is described as a discrete part of the LCLC that serves to prevent postero lateral rotatory instability and varus stress [5].

O'Driscoll et al [6] anatomically defined this LUCL from its common insertion with the LCLC on the lateral epicondyle to its isolated insertion at the tubercle on the supinator crest. The function of the LCLC and the importance of each component and the surrounding soft tissues and the mechanism of poster lateral rotatory instability (PLRI) continue to be investigated and debated. Additional stability is conferred by dynamic structures-the muscles crossing the elbow joint [6].

Recent studies continue to develop our understanding of the role and importance of the primary and secondary stabilizers of the elbow.

Disruption or attenuation of LCL structures is associated with elbow dislocation, varus stress, and iatrogenic causes and patients may present with complaints of catching, clicking, instability, such as Posterolateral rotatory instability (PLRI), or recurrent dislocation

Dislocation of the previously stable elbow, for example, while trying to get out of bed is a rather unusual mechanism. It might suggest that the elbow had a degree of instability but the patient denied having any significant problems with the elbow prior to the incident.

In addition it might be expected that the patient would have generalised ligamentous laxity but our patient did not have any laxity according to Beighton's criteria [7] for ligamentous laxity.

Elbow dislocation has been described in a child treated with overhead traction for supracondylar fracture after closed reduction [8]. In this case the anterior capsular rupture during the time of fracture was thought to be the precipitating factor while on traction. However in our case the anterior capsule would not have been acutely disrupted.

Rasool et al reviewed 33 children with dislocated elbow with a mean of 10 months (4 to 48) and found no case of recurrence indicating that children do well following this type of injuries [1].

## Conclusion

Our patient is of interest as there is no previously reported case in the literature of such a late low energy dislocation. It suggests that in children if there is a failure of the capsule and ligamentous structures to become correctly reattached after traumatic dislocation it may predispose to further dislocation from low energy trauma. It certainly demonstrates that even in the absence of instability a patient can be predisposed to low energy dislocation of the elbow.

## Abbreviations

PLRI – Poster lateral rotatory instability; LCLC – Lateral collateral ligament complex; LUCL – Lateral ulnar collateral ligament

## Competing interests

No benefits in any form have been received or will be received from a commercial party related directly or indirectly to the subject of this article

## Authors' contributions

KP - reporting the case, including literature search. PC - Consultant (Upper limb specialist) who diagnosed and reduced the elbow. DM - Supervising consultant. All authors read and approved the final manuscript.

## Consent

Written informed consent was obtained from the patient for publication of this Case report and any accompanying images. A copy of the written consent is available for review by the Editor-in-Chief of this journal.
